# *Spirulina platensis* Reduced Oxidative Damage Induced by Chlorpyrifos Toxicity in Nile Tilapia (*Oreochromis niloticus*)

**DOI:** 10.3390/ani10030473

**Published:** 2020-03-12

**Authors:** Mohamed M. Abdel-Daim, Mahmoud A.O. Dawood, Mohamed Elbadawy, Lotfi Aleya, Saad Alkahtani

**Affiliations:** 1 Department of and Zoology, College of Science, King Saud University, P.O. Box 2455, Riyadh 11451, Saudi Arabia; 2 Pharmacology Department, Faculty of Veterinary Medicine, Suez Canal University, Ismailia 41522, Egypt; 3 Department of Animal Production, Faculty of Agriculture, Kafrelsheikh University, Kafrelsheikh 33516, Egypt; 4 Department of Pharmacology, Faculty of Veterinary Medicine, Benha University, Moshtohor, Toukh, Elqaliobiya 13736, Egypt; 5 Chrono-Environment Laboratory, UMR CNRS 6249, Bourgogne Franche-Comté University, F-25030 Besançon CEDEX, France

**Keywords:** chlorpyrifos, *Spirulina platensis*, Nile tilapia, oxidative stress, blood biochemistry

## Abstract

**Simple Summary:**

Chlorpyrifos (CPF) is one of the popular crop pests that is widely used in the agriculture sector. In the aquaculture sector, several strategies were applied to mitigate the negative impacts of CPF on aquatic animals through the application of feed additives. *Spirulina platensis* (SP) is well reported as a functional feed additive to enhance the physiological, immunological, and anti-oxidative status in farmed fish. In the current study, fish were randomly stocked in five groups where the first and second groups reared in clean water without CPF toxicity and were fed the basal diet without SP or with SP at 1%, respectively. Meanwhile, the third, fourth, and fifth groups were exposed to CPF (15 μg/L) in rearing water and fed SP at 0, 0.5, and 1%, respectively. Under CPF exposure, SP displayed preventive and restorative impacts against toxicity in Nile tilapia. Fish fed SP-supplemented diet showed decreased alanine aminotransferase (ALT), aspartate aminotransferase (AST), alkaline phosphatase (ALP), cholesterol, urea, and creatinine as well as increased total protein, albumin, superoxide dismutase (SOD), and catalase (CAT) activities. The outcomes suggested that SP is efficient in protecting Nile tilapia from CPF toxicity by increasing the antioxidative response.

**Abstract:**

Due to the numerous pharmacological impacts of *Spirulina platensis* (SP), the effects of SP on the oxidative status of Nile tilapia farmed under chlorpyrifos (CPF) ambient toxicity were considered in this study. Fish (60 ± 6.1 g) was randomly stocked in five groups where the SP free diet was fed to the control group while the second one was fed 1% SP without CPF exposure. Additionally, CPF (15 μg/L) was added in water and fish were fed with SP at 0, 0.5, and 1% (third, fourth, and fifth groups, respectively). Samples of blood and gills, kidneys, and liver tissues were assayed for biochemical measurements. Fish exposed to CPF exhibited significant (*p* ≤ 0.05) increments of serum alanine aminotransferase (ALT), aspartate aminotransferase (AST), alkaline phosphatase (ALP), cholesterol, urea, creatinine, and malondialdehyde (MDA), while significantly decreased total protein, albumin, and antioxidative enzyme activities (superoxide dismutase (SOD) and catalase (CAT) were observed in tilapia exposed to CPF (*p* ≤ 0.05). In contrast, SP feeding resulted in decreased levels of ALT, AST, ALP, cholesterol, urea, and creatinine as well as increased total protein, albumin, SOD, and CAT activities. Based on the obtained results, it can be suggested that SP is efficient in protecting Nile tilapia from CPF toxicity by increasing the antioxidative response.

## 1. Introduction

There are several environmental risks facing the aquaculture industry, including water toxicity, which weakens the immunity of fish and allows the spread of infectious diseases [1]. Pesticides are among the major water pollutants due to their increased application in the agriculture sector. Fish can be considered as a natural detector for direct pesticide accumulations in water which indirectly can be accumulated in the human body [2,3]. This has raised great concerns on the safety of food and food supplies on human health [4,5]. Pesticides can impair the health status of fish via anemia, hydromineral disequilibrium, neurotoxicity, and histopathological damage [6,7].

Chlorpyrifos (CPF) is one of the popular crop pests that is used widely in the agriculture sector [8]. Due to the limitations of freshwater sources, drainage water can be recycled and reused in fish farms. Consequently, several toxins and unfavorable residues can be accumulated in the fish body and result in severe direct or indirect impacts [4,9]. CPF toxicity impaired the immune and antioxidative responses in aquatic animals through the excessive lipid peroxidation, apoptosis, and neurobehavioral development [5]. As a result, fish susceptibility to attack infectious diseases increased with induced inflammation. CPF toxicity resulted in impaired physiological and antioxidative responses in Caspian brown trout (*Salmo trutta caspius*) [10], Chinook salmon (*Oncorhynchus tshawytscha*) [11], *Catla catla*, *Labeo rohita*, *Cirrhinus mrigala*, *Cyprinus carpio* [12,13], Mosquito fish (*Gambusia affinis*) [14], Nile tilapia (*Oreochromis niloticus*) [4], and Guppy fish (*Poecilia reticulata*) [15].

*Spirulina platensis* (SP) is reported to alleviate the physiological, immunological, and anti-oxidative status in farmed fish [16]. SP has many functional and bioactive substances, such as vitamins, minerals, carotenoids, polysaccharides, and γ-linolenic acid, which make it among the healthiest food additives [17,18,19,20]. It has been reported that SP displayed preventive and restorative roles against lipid peroxidation and oxidative damage in Nile tilapia exposed to deltamethrin [21]. However, until now, there is no report investigating the impacts of dietary SP on Nile tilapia exposed to CPF. Hence, the current investigation was hypothesized to reveal the functional role of SP on Nile tilapia under ambient toxicity of CPF via evaluating the renal, hepatic, and oxidative responses.

## 2. Materials and Methods

### 2.1. Fish, Diet, Chlorpyrifos (CPF), and Experimental Design

The experimental procedure was approved by the Faculty of Agriculture, Kafrelsheikh University, Egypt (Approval No. 07-2018). Tilapia fish were collected from a local farm (Kafrelsheikh, Egypt). The fish were then acclimated to lab conditions for 14 days. Then, fish (60 ± 6.1 g) were allocated into ten glass aquaria (40 × 60 × 70 cm, 10 fish per each). The aquaria were equipped with continuous aeration and half of the water was daily replaced with freshly dechlorinated water.

The basal diet (Table 1) was prepared as described before [22] and fed to fish for 28 days. The fish were randomly stocked in five groups (2 aquaria/group, duplicates) where the SP free diet was fed to the control group while the second group was fed the basal diet supplemented with 1% SP (HerbaForce, Lewes, UK) without CPF (48%, El Nasr chemical Co., Abu-Rawash, Egypt) exposure. Additionally, CPF (15 μg/L) was added as indicated by Oruc [23] to water and fish were fed the basal diet supplemented with SP at 0, 0.5, and 1% (third, fourth, and fifth groups, respectively). The glass aquaria were siphoned daily to remove the feces and remaining diets and refilled with CPF mixed water. The CPF stock was prepared 24 h before siphoning in a separate tank (200 L) with a concentration of 15 μg/L [21,24]. After the siphon, the same amount of removed water was replaced with CPF-mixed water from the stock tank to keep the proposed concentration of CPF fixed during the trial. The fish were fed the test diets twice a day (8:00 and 16:00) up to the satiation level. Water quality items were fixed during the trial to be dissolved oxygen—DO (6.5 ± 0.5 mg/L), total ammonia (0.21 ± 0.01 mg/L), pH (7.1 ± 0.8), temperature (25 ± 2 °C), and photoperiod (12 h light:12 h dark). The fish were visually checked on a regular basis during the trial.

### 2.2. Sampling

The fish were euthanized (0.02% benzocaine) and their blood was gathered from the caudal vein (5 fish/aquarium), left for 2 h at room temperature, and the serum was collected by a centrifuging at 6000× *g* for 15 min at 4 °C. Serum samples were kept at −30 °C until analysis.

Gills, kidneys, and liver tissues were dissected (5 fish/aquarium) and immediately perfused with cold physiological saline (PSA, NaCl 0.9%). The tissues were then homogenized, filtered, and centrifuged at 6000× *g* for 20 min at 4 °C. The supernatant was collected and stored at −80 °C for further analysis.

### 2.3. Biochemical Measurements

The supernatant protein content was estimated as mg protein/g of wet tissue [25]. Superoxide dismutase (SOD), catalase (CAT), and malondialdehyde (MDA) were estimated as reported before ([26], [27], and [28], respectively).

Diagnostic commercial kits (Biodiagnostic Co., Dokki-Giza, Egypt) were used for alanine aminotransferase (ALT) and aspartate aminotransferase (AST), alkaline phosphatase (ALP), cholesterol, total serum protein (sTP), albumin, urea, and creatinine analysis according to the manufacturer’s protocol. The analytical procedures were applied by using 8 replicates for each treatment.

### 2.4. Statistical Analysis

Before running the statistical analysis, all data were examined for the normality and homogeneity by the Shapiro–Wilks test. All statistical differences were evaluated by a one-way ANOVA test with Duncan post-hoc. The records were displayed as means ± standard error (SE) and considered significantly different at *p* ≤ 0.05. The SPSS software was used during statistical analysis (version 22, SPSS Inc., Chicago, IL, USA).

## 3. Results

### 3.1. Blood Biochemical Markers

Fish exposed to CPF without SP feeding displayed significantly (*p* ≤ 0.05) higher AST, ALT, and ALP activities versus fish that were fed the diet with SP 1% alone and the control group. Fish under CPF toxicity and fed SP at 1% displayed lower AST, ALT, and ALP than fish fed SP at 0.5% (*p* ≤ 0.05) without differences with the control group (*p* ≥ 0.05) (Table 2).

Levels of sTP and albumin displayed higher values in tilapia fed with SP versus the control (*p* ≤ 0.05) (Table 2). However, fish exposed to CPF showed reduced sTP and albumin levels versus the control and SP groups. Under CPF toxicity, tilapia fed with the basal diet without SP displayed lower sTP and albumin levels than fish fed SP at 0.5% or 1% levels (*p* ≤ 0.05).

According to Table 2, fish exposed to CPF exhibited significantly higher cholesterol levels versus the control group (*p* ≤ 0.05). Further, both groups of fish exposed to CPF and fed with SP showed either higher (0.5% SP) or comparable (1% SP) cholesterol levels when matched with the control group. Similarly, tilapia exposed to CPF revealed higher urea and creatinine levels than tilapia fed with the control diet or SP at 1% without CPF toxicity (*p* ≤ 0.05). Under CPF toxicity, fish fed with SP at 0.5% or 1% levels displayed lower urea and creatinine levels than fish fed with the basal diet without SP. The levels of urea and creatinine were lower in tilapia fed with SP at 1% than tilapia fed with 0.5% under CPF toxicity, indicating a dose-dependent effect.

### 3.2. Tissue Lipid Peroxidation

**Figure 1:**
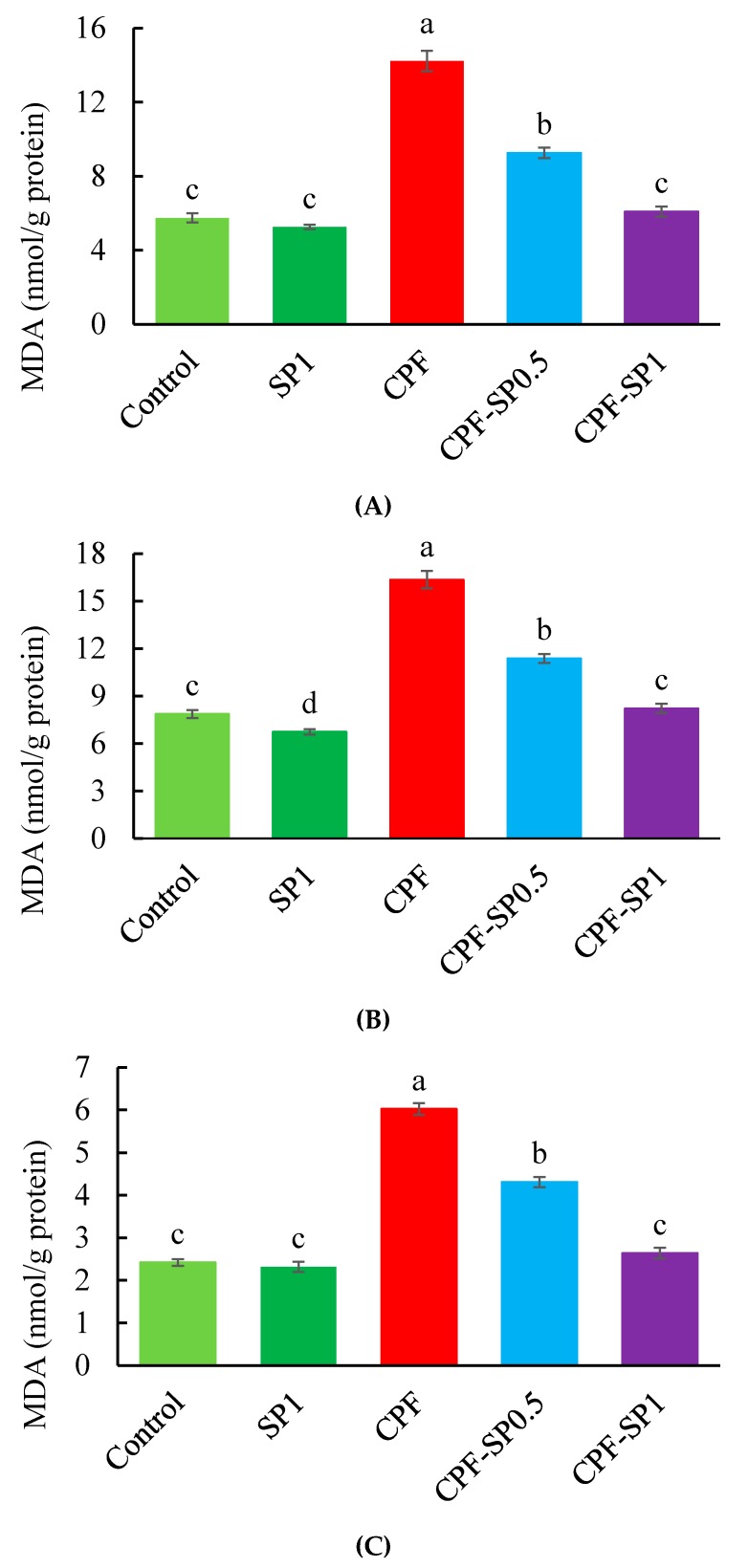
(**A**) Liver, (**B**) kidney, and (**C**) gill malondialdehyde (MDA) levels in the treated groups (nmol/g protein). Data are expressed as means ± SE (*n* = 8). Bars with different letters are significantly different (*p* ≤ 0.05).

CPF induced the lipid peroxidation in tilapia by displaying significantly high levels of MDA in liver, kidneys, and gills over the control (*p* ≤ 0.05) (Figure 1). Without CPF toxicity, SP inclusion in tilapia feeds markedly reduced the MDA levels compared with the control group. Furthermore, fish exposed to CPF showed higher MDA in liver, kidneys, and gills than CPF-intoxicated groups fed with SP at 0.5 or 1% levels (Figure 1).

### 3.3. Tissues Enzymatic Antioxidant Biomarkers

**Figure 2:**
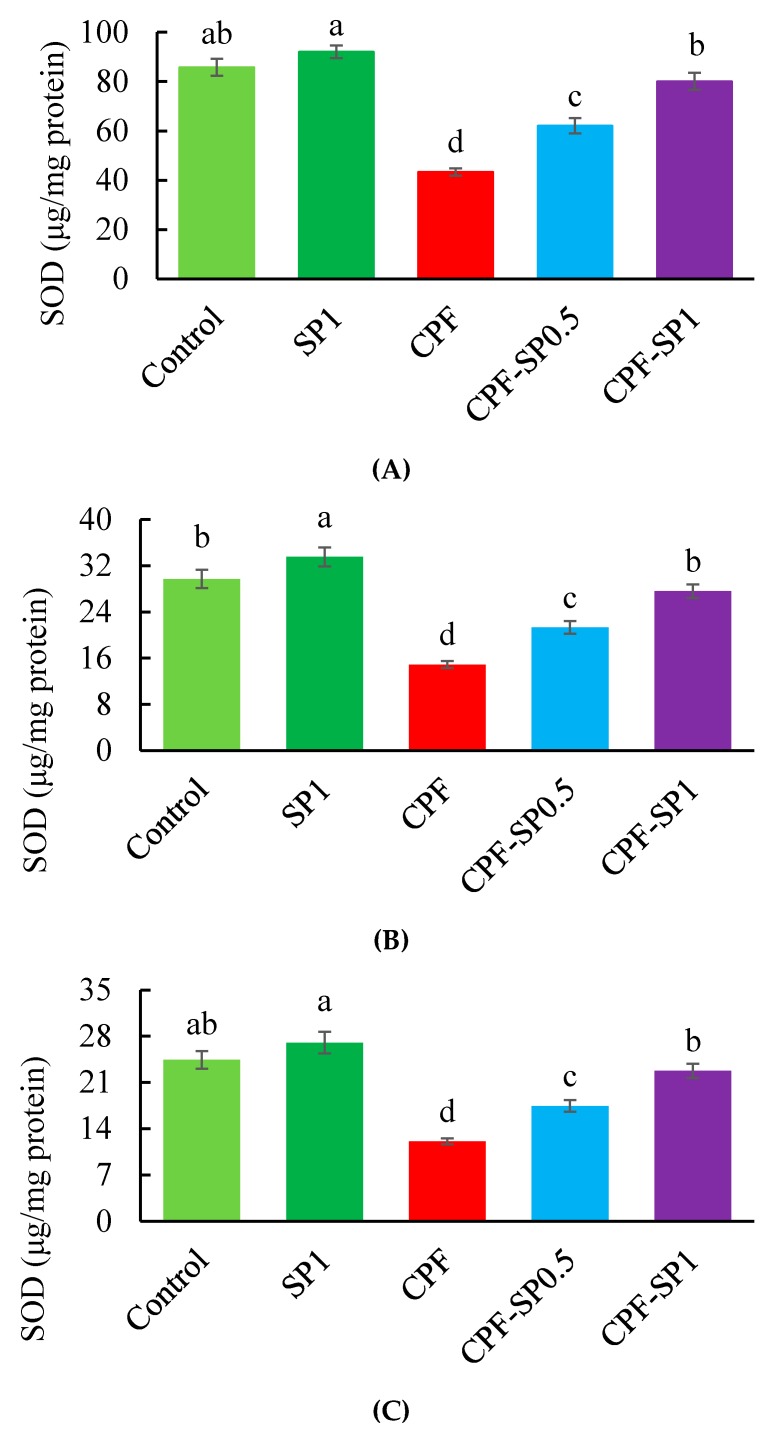
(**A**) Liver, (**B**) kidney, and (**C**) gill superoxide dismutase (SOD) levels in the treated groups (μg/mg protein). Data are presented as means ± SE (*n* = 8). Bars with different letters are significantly different (*p* ≤ 0.05).

**Figure 3:**
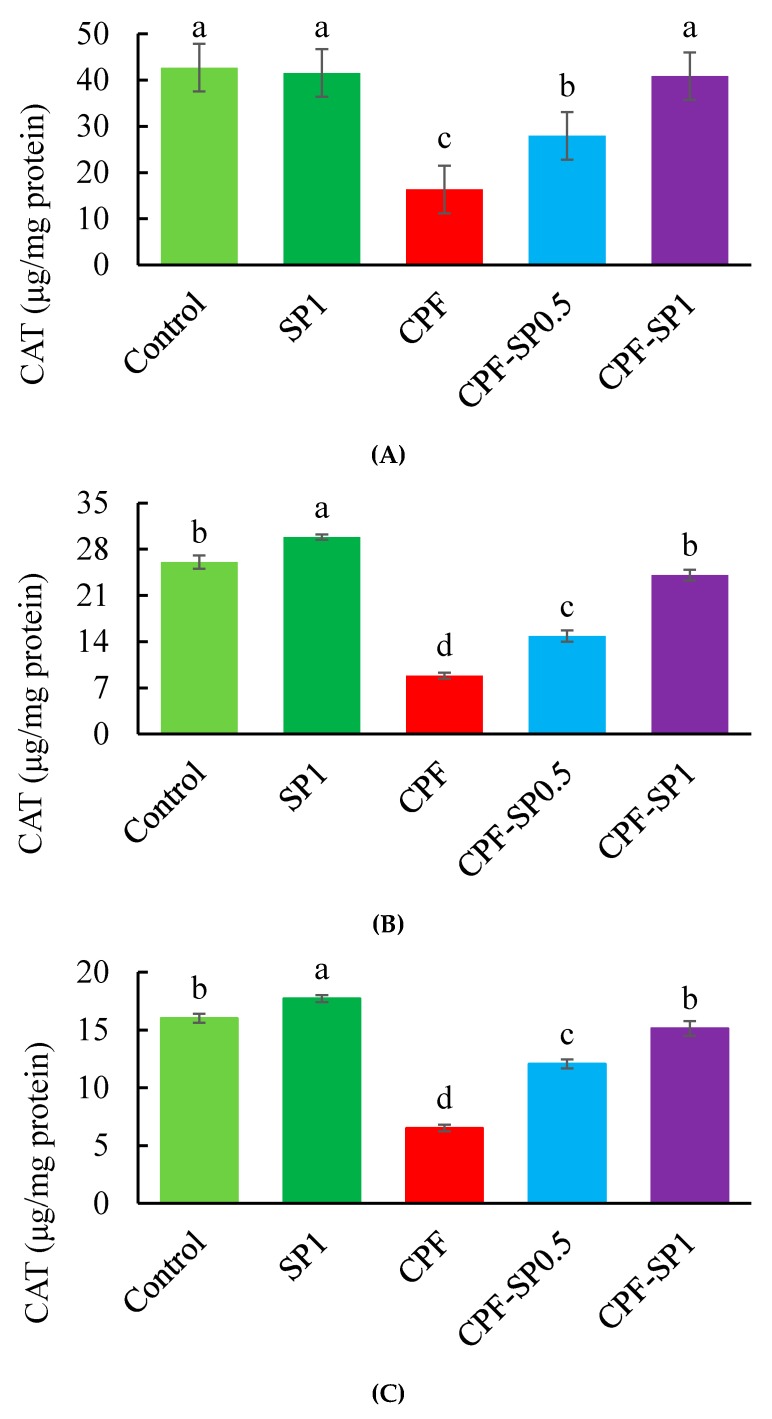
(**A**) Liver, (**B**) kidney, and (**C**) gill catalase (CAT) levels in the treated groups (μg/mg protein). Data are presented as means ± SE (*n* = 8). Bars with different letters are significantly different (*p* ≤ 0.05).

Results of antioxidant biomarkers (SOD and CAT) are presented in Figure 2 and Figure 3, respectively. Fish exposed to CPF exhibited lower SOD and CAT than the control and SP groups without CPF toxicity (*p* ≤ 0.05). Under CPF toxicity, tilapia fed with SP at 0.5% or 1% levels displayed higher SOD and CAT levels than fish fed the basal diet without SP. The levels of SOD and CAT were higher in tilapia fed with SP at 1% than tilapia fed with 0.5% under CPF toxicity. In non-CPF-intoxicated groups, CAT showed increased activities in kidney and gills tissues of fish fed with SP (Figure 3B,C), while no alterations were stated in the case of liver CAT (*p* > 0.05) (Figure 3A).

## 4. Discussion

Chlorpyrifos is a wide-spectrum pesticide of organophosphate type that is greatly used for domestic and agricultural objects [15]. The toxic influences of CPF are progressively threatening humans and the life of aquatic animals showing several physiological and immunological impacts [10]. CPF toxicity can induce neurobehavioral development by inhibiting the acetylcholinesterase once metabolically activated within the organism [29]. However, the application of functional feed additives can protect fish from the impacts of pesticides, insecticides, and xenobiotics via stimulating the immune and antioxidative responses [30,31,32].

In the present study, the possible role of SP in the attenuation of CPF-induced toxic effects of Nile tilapia has been evaluated in which fish were fed with a diet supplemented with SP at a level of 0.5% and 1% for 28 successive days. The results of this experiment showed a substantial neutralization of CPF toxic effects in the SP-fed fish group, whereas there was a pronounced reduction of tissues (liver, kidney, and gills) MDA as well as blood ALT, AST, and ALP levels, with improvements of CAT and SOD activities. Moreover, it considerably caused an improvement of sTP and albumin levels. The functionality of SP is due to its rich content of functional compounds, such as C-phycocyanins, β-carotene, minerals, and vitamins, which have immunomodulatory and antioxidative roles [33].

The blood biochemical variables showed the condition of the renal (creatinine and urea) and hepatic (ALT, AST, and ALP) organs of tilapia exposed to ambient CPF and fed diets with or without SP. Fish exposed to CPF without SP feeding displayed higher AST, ALT, and ALP activities than fish fed with SP. The obtained results indicate that the hepatic tissue of fish was severely impaired by CPF exposure while SP represented a functional remedy for fish. In the same way, ALT, AST, and ALP displayed high activities in fish exposed to CPF as explained before by Adel et al. [10] and Jaffer et al. [34]. For a long time, SP has been used as a nutraceutical or functional food additive for both human and aquatic animals [20]. 

As renal dysfunction markers, blood creatinine and urea displayed increased values in tilapia exposed to CPF, whereas SP alleviated the toxicity of CPF. The increased creatinine and urea can be attributed to the diminishing effect of CPF on glomerular filtration and protein catabolic rates, which in turn can decrease the ability of the kidney to get rid of urea and urine [35,36]. Similarly, Nile tilapia exposed to CPF or other pesticides revealed increased serum levels of urea and creatinine [21,37], while other studies showed reduced levels of them with the inclusion of feed additives in fish diets [8,30].

CPF also increased the level of total cholesterol in tilapia, while feeding with SP diminished its level. The high levels of cholesterol in the blood normally refer to the high metabolic rates of lipids in body tissues especially under stressful conditions [38]. However, the results of the present study are further confirming the role of SP in maintaining the normal healthy condition of tilapia exposed to CPF by reducing the level of blood cholesterol [39]. Similarly, SP feeding reduced the harsh effects of diazinon on tilapia by lowering the level of blood cholesterol [37].

Blood albumin and sTP levels are normally used as humoral immunity indicators [40]. In the current study, levels of albumin and sTP displayed increased values in fish fed with SP while fish exposed to CPF showed reduced albumin and sTP levels. Further, fish exposed to CPF without SP feeding exhibited lowered albumin and sTP levels than fish fed with SP. The decreased level of sTP can be attributed to the vascular leaking and high proteolysis rate [41], while the decreased albumin can be interpreted by the high rate of renal excretion and failure of protein synthesis due to liver malfunction in fish exposed to CPF [34,42].

Oxidative stress is the abnormal or overproduction of reactive oxygen radicals (ROS) to the level that beats the endogenous protective mechanisms of the body and in turn damages the cellular components [43,44,45]. The oxidative stress will lead to a state of inequality between production and removal of ROS in the cell, which makes ROS attack the cellular components (lipids, proteins, and DNA) causing lipid peroxidation of the cell membrane, mitochondrial dysfunction, and DNA fragmentation, which finally terminates with cell death [33]. The high levels of lipid peroxidation can be expressed by the MDA [46]. CPF induced the lipid peroxidation in tilapia by increased MDA levels in gills, kidneys, and liver, while SP inclusion in tilapia feeds markedly reduced the MDA levels in the current study.

There are several defense mechanisms of the body to maintain the redox homeostasis and normalize the cell functions through the attenuation of ROS by various antioxidants, such as SOD and CAT [47,48,49]. Results of the current study revealed that fish exposed to CPF exhibited low SOD and CAT activities, while SP alleviated the activities of SOD and CAT compared with fish exposed to CPF. It was demonstrated that the aquatic toxicity caused by the waterborne pollutants will lead to ROS production, which can trigger the oxidative damage and death of the living aquatic organisms [4]. In the present study, the toxic impacts induced by CPF on the general condition of Nile tilapia can be attributed to the damages that occurred in gills. Gills are directly exposed to CPF toxicity in rearing water and this could imbalance the gas exchange ratio initiating osmoregulation and nitrogenous excretion disorders [21]. It has been shown that, regardless of the potential cytotoxic influences of ROS, cells have a wide range of defense mechanisms that help to attenuate the injurious impacts of these radicals. Supplementation of SP in Nile tilapia diets exhibited protective effects through the removal of excess ROS, thus resulting in better antioxidant capacity. It can be assumed that SP supplementation resulted in an induced antioxidative response either by the improvement of the action of the SOD and CAT enzymes or by removing the extra ROS produced and accordingly avoiding lipid peroxidation (MDA increase) due to its content of phycocyanin and carotenoids [33]. SP has a high content of β-carotene which can scavenge ROS molecules including “peroxyl, alkoxyl, and hydroxyl radicals” besides its role in the reduction of nitrite and nitric oxide synthase to minimize the liver lipid peroxidation [50,51,52]. 

*Spirulina* represented an effective strategy due to its nutritional composition and bioactive compounds [16]. Dietary SP supplementation reduced the blood ALT, AST, ALP, urea, creatinine, and cholesterol levels. In the meantime, it lowered the lipid peroxidation (MDA) in the tested tissues besides its role in the elevation of sTP, albumin, and enzymatic antioxidant (SOD and CAT) activities.

## 5. Conclusions

To sum up, the inclusion of SP in the tilapia diet at 0.5–1% is a good choice to protect against biohazards induced by CPF toxicity in Nile tilapia.

## Figures and Tables

**Table 1:** Components and chemical analysis of the basal diet.

Ingredients	Percentage (%)
Fish meal	26
Yellow corn	29
Soybean meal	20.5
Corn gluten meal	2
Wheat bran	9
Egyptian clover meal	7.2
Cod liver oil	3
Gelatin	2
Vitamin mixture	0.5
Mineral mixture	0.5
Salt	0.3
**Chemical analysis**	
Crude protein %	31.78
Ether extract %	7.15
Ash %	8.14

Trace minerals and vitamin mixtures were supplemented as endorsed by Abdel-Daim et al. [22].

**Table 2:** Blood biochemical parameters in the examined groups *.

Parameters	Experimental Groups				
	Control	SP1	CPF	CPF-SP0.5	CPF-SP1
AST (U/L)	40.7 ± 2.16^cd^	39.2 ± 2.21^d^	99.4 ± 1.45^a^	71.3 ± 2.08^b^	47.4 ± 2.1^c^
ALT (U/L)	15.4 ± 0.56^c^	15.3 ± 0.58^c^	35.4 ± 1.41^a^	24.8 ± 0.57^b^	16.9 ± 0.76^c^
ALP (U/L)	9.81 ± 0.55^cd^	8.36 ± 0.40^d^	20.9 ± 0.89^a^	14.3 ± 0.40^b^	10.1 ± 0.36^c^
Total protein (g/dL)	5.24 ± 0.35^b^	5.99 ± 0.18^a^	3.59 ± 0.17^d^	4.42 ± 0.25^c^	4.99 ± 0.28^bc^
Albumin (g/dL)	3.26 ± 0.20^b^	3.77 ± 0.11^a^	2.04 ± 0.090^d^	2.64 ± 0.20^c^	3.21 ± 0.20^b^
Cholesterol (mg/dL)	226.7 ± 9.15^c^	220.6 ± 6.26^c^	344.2 ± 14.7^a^	279.7 ± 3.04^b^	238.5 ± 6.92^c^
Urea (mg/dL)	7.02 ± 0.44^c^	6.74 ± 0.23^c^	17.7 ± 0.86^a^	11.5 ± 0.23^b^	7.63 ± 0.41^c^
Creatinine (mg/dL)	0.51 ± 0.03^c^	0.46 ± 0.020^c^	2.57 ± 0.21^a^	1.28 ± 0.05^b^	0.67 ± 0.04^c^

* Data are presented as means ± SE (*n* = 8), values with different superscripts in the same row are significantly different (*p* ≤ 0.05). Aspartate aminotransferase (AST), alanine aminotransferase (ALT), alkaline phosphatase (ALP), *Spirulina platensis* at 1% (SP1), *Spirulina platensis* at 0.5% (SP0.5), chlorpyrifos (CPF).
